# Design of a Hybrid Refractive/Diffractive Lens System for Broadband UV

**DOI:** 10.3390/s23010143

**Published:** 2022-12-23

**Authors:** Yuan Hu, Jiaqi Huo, Binpeng Cheng

**Affiliations:** School of Optoelectronic Engineering, Changchun University of Science and Technology, Changchun 130012, China

**Keywords:** UV broad spectrum, double-layer diffractive optical elements, diffraction efficiency, refractive/diffractive hybrid lens system design

## Abstract

Traditional broadband UV optical designs often have complex structural problems and cannot meet the current requirements of light and miniaturization. In this study, we first design the substrate material of double-layer diffractive optical elements (DOEs) in the 0.23–0.8 μm band, optimize the diffraction efficiency and analyze the effect of the angle of incidence on the diffraction efficiency of double-layer DOEs. Second, we design a refractive lens system and a refractive/diffractive hybrid lens system with double-layer DOEs designed for a wide UV wavelength range of 0.23–0.8 μm, a half field of view of 15 mm, an objective aperture of 0.1 and a magnification of 1. The refractive/diffractive hybrid lens system designed with seven lenses produces a higher image quality than the refractive lens system. The novel design is an effective solution to the problem of the low transmission rate of traditional UV refractive lens systems.

## 1. Introduction

Lens system imaging is used to obtain information about a target, and extending the imaging spectrum is an important way of obtaining more information. Refractive lens systems [1,2,3] used in the broadband UV range are often designed with multiple lenses or by using coated prisms to ensure the lens system meets the requirements for broadband operation. However, the low UV transmittance of glass reduces the overall transmission rate of the refractive lens system, and the use of prisms increases the weight of the lens system and the difficulty of installation, which is not conducive to fabricating a lightweight miniaturized lens system. Although reflective optical systems [4,5] can meet the requirements of UV broadband operation, their field of view is limited to a small range of object heights and 0.8°. Current designs of hybrid refractive/diffractive lens systems [2,6,7,8] with single-layer diffractive optical element (DOE) in the UV band are only applicable to narrow wavelengths. This narrow operating band notwithstanding, the introduction of single-layer DOE into a lens system provides an ideal way to achieve lightweight miniaturized UV wide-spectrum large-field lens systems.

**Figure 1:**
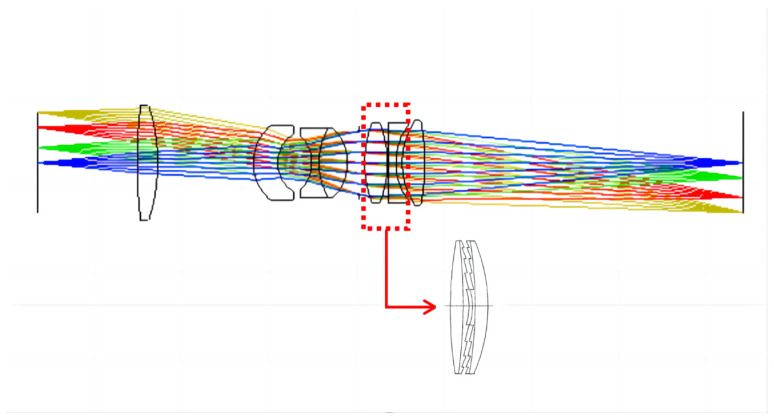
Refractive/diffractive hybrid lens system with double-layer DOEs.

Double-layer DOEs are more suitable for broad bands than single-layer DOE and can be used in lens systems to meet the requirements of the UV broad-spectrum large field-of-view operation and realize lighter miniaturized lens systems. In this study, we analyze and optimize the bandwidth integration average diffraction efficiency (BIADE) of double-layer DOEs in the 0.23–0.8 μm band and fabricate a substrate material for double-layer DOEs for use in a lens system. The performance of a UV broad-spectrum refractive/diffractive hybrid lens system with double-layer DOEs (as shown in Figure 1 below) is compared with that of the traditional refractive lens system. The double-layer-DOE hybrid system outperforms the refractive system. The feasibility of using double-layer DOEs in UV broad-spectrum lens systems provides a means of miniaturizing a lightweight system.

## 2. Analysis and Optimization of Double-Layer Diffractive Optical Elements for a Broad Ultraviolet Spectrum

The diffraction efficiency of a single-layer DOE is 100% only at the design wavelength. As the diffraction efficiency drops rapidly at wavelengths other than the design wavelength, a nonworking-order diffraction beam can cause the contrast ratio in the imaging plane to decrease; thus, the single-layer DOEs that are primarily used in the lens systems operate within a narrow waveband. In contrast, double-layer DOEs are based on different dispersive materials and different surface relief heights. Double-layer DOEs can be an effective means of achieving high diffraction efficiency in wide wavebands. A schematic of a double-layer DOE is shown below.

**Figure 2:**
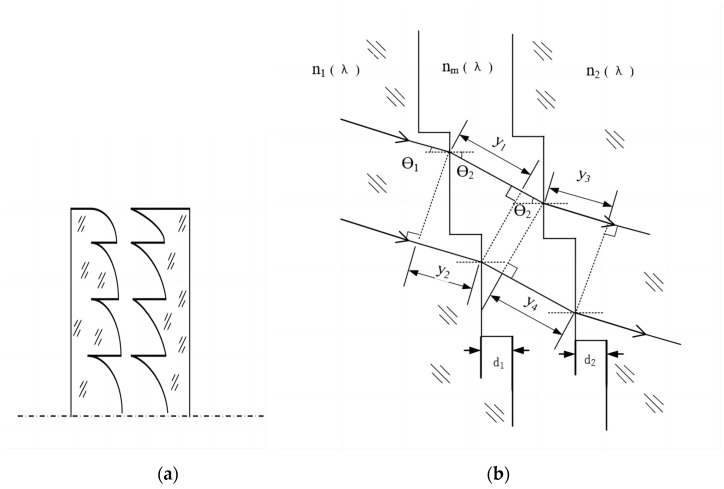
Schematic of a double-layer DOEs: (**a**) structure; (**b**) operating principle, showing light incident at an angle *θ*_1_ on the internal structure.

For a parallel beam that is vertically incident on a double-layer DOE (angle of incidence $\theta_{1}=0$), the m-order diffraction efficiency ($\eta_{m}$) can be determined using Equation (1), as follows [9]:

(1) $$\eta_{m}=\sin c^{2}\left\{m-\frac{d_{1}\left[1-n_{1}\left(\lambda \right)\right]+d_{2}\left[n_{2}\left(\lambda \right)-1\right]}{\lambda }\right\}$$

where $n_{1}\left(\lambda \right)$ and $n_{2}\left(\lambda \right)$ are the refractive indexes of the two DOEs at the wavelength λ, and $d_{1}$ and $d_{2}$ are the corresponding microstructure heights, as shown in Figure 2b.

The BIADE of DOEs operating in a specific waveband range is

(2) $${\bar{\eta }}_{m}\left(\lambda \right)=\frac{1}{\lambda_{\max}-\lambda_{\min}}\int_{\lambda_{\min}}^{\lambda_{\max}}\eta_{m}d\lambda$$

where $\lambda_{\min}$ and $\lambda_{\max}$ denote the minimum and maximum wavelength values in the operating band range of DOEs, respectively.

The following relationship holds for two different dispersion materials and two design wavelengths, $\lambda_{1}$ and $\lambda_{2}$, in the dual design band.

(3) $$\begin{array}{l} \left\{ \begin{array}{l} d_{1}\left[1-n_{1}\left(\lambda_{1}\right)\right]+d_{2}\left[n_{2}\left(\lambda_{1}\right)-1\right]=m\lambda_{1}\\ d_{1}\left[1-n_{1}\left(\lambda_{2}\right)\right]+d_{2}\left[n_{2}\left(\lambda_{2}\right)-1\right]=m\lambda_{2} \end{array}\right.\\ d_{1}=\frac{m\lambda_{2}\times \left[n_{2}\left(\lambda_{1}\right)-1\right]-m\lambda_{1}\times \left[n_{2}\left(\lambda_{2}\right)-1\right]}{\left[1-n_{1}\left(\lambda_{2}\right)\right]\times \left[n_{2}\left(\lambda_{1}\right)-1\right]-\left[1-n_{1}\left(\lambda_{1}\right)\right]\times \left[n_{2}\left(\lambda_{2}\right)-1\right]}\\ d_{2}=\frac{m\lambda_{2}\times \left[1-n_{1}\left(\lambda_{1}\right)\right]-m\lambda_{1}\times \left[1-n_{1}\left(\lambda_{2}\right)\right]}{\left[n_{2}\left(\lambda_{2}\right)-1\right]\times \left[1-n_{1}\left(\lambda_{1}\right)\right]-\left[n_{2}\left(\lambda_{1}\right)-1\right]\times \left[1-n_{1}\left(\lambda_{2}\right)\right]} \end{array}$$

The diffraction efficiency of a double-layer DOE is not uniformly distributed over the entire waveband and must therefore be optimized. The diffraction efficiency at long wavelengths needs to be equal to that at short wavelengths, as follows:

(4) $$\eta_{m}\left(\lambda_{S}\right)=\eta_{m}\left(\lambda_{L}\right)$$

Combining Equations (1) and (4) yields

(5) $$\frac{d_{2}}{d_{1}}=\frac{\left[n_{1}\left(\lambda_{S}\right)-1\right]\cdot \lambda_{L}-\left[n_{1}\left(\lambda_{L}\right)-1\right]\cdot \lambda_{S}}{\left[n_{2}\left(\lambda_{S}\right)-1\right]\cdot \lambda_{L}-\left[n_{2}\left(\lambda_{L}\right)-1\right]\cdot \lambda_{S}}$$

**Figure 3:**
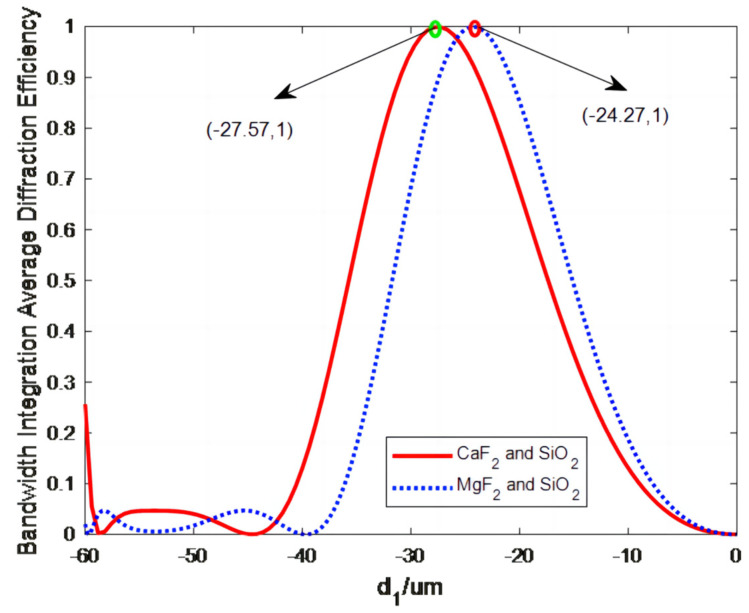
Relationship between BIADE and the microstructure height d_1_ of double-layer DOEs.

Substituting relevant data into Equation (5) yields a relationship between d_2_ and d_1_. m = +1; generally, the +1 order diffraction beam is taken as the imaging beam. Commonly available materials with high transmittance in the wavelength range of 0.23–0.8 μm and facile processability include SiO_2_, CaF_2_, and MgF_2_, of which any two can be combined for use as a DOE substrate. The microstructure height is a major parameter for DOE processing. When using Equation (3) to estimate the material dispersion coefficients, two materials with highly similar Abbe numbers make the denominator small, which leads to a large microstructure height, d_1_. A large microstructure height for multilayer DOEs makes processing difficult and leads to a low diffraction efficiency [10,11]. Therefore, the similar dispersion coefficients of CaF_2_ and MgF_2_ led to the exclusion of this combination of glasses for substrate fabrication. Substituting the aforementioned relationship between d_1_ and d_2_ into Equations (1) and (2) yields the relationship between the microstructure height d_1_ and BIADE of double-layer DOEs, as shown in Figure 3 for three substrates of different material pairs.

**Figure 4:**
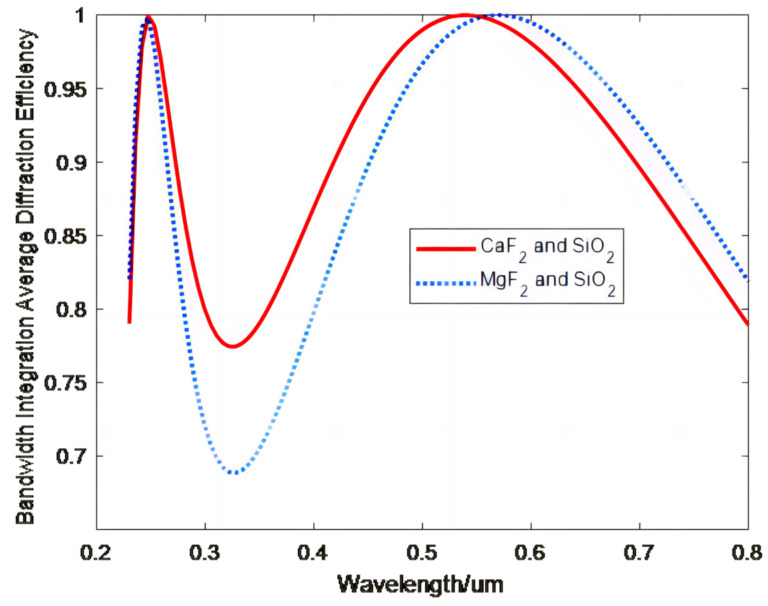
BIADE versus the wavelength after optimization of double-layer DOEs for substrates consisting of different material combinations.

The optimization process yielded a combined microstructure height of −27.57 μm for CaF_2_ and SiO_2_ and −24.27 μm for MgF_2_ and SiO_2_ for a 100% diffraction efficiency. Figure 4 shows the relationship between the wavelength and BIADE obtained for three substrates consisting of different material combinations for the optimized double-layer DOEs.

Figure 4 shows that the highest BIADE in the wavelength range of 0.23–0.8 μm of the double-layer DOE is obtained using a substrate consisting of CaF_2_ and SiO_2_. Therefore, CaF_2_ and SiO_2_ were chosen as the substrate materials for the subsequent design of double-layer DOEs.

The previous analysis was based on orthogonal incidence, where the heights of the two optimized microstructures were found to be $d_{1}=-27.57\;\mu m$ and $d_{2}=-24.89\;\mu m$, respectively. Next, we consider the effect of the angle of incidence on the diffraction efficiency in the case of oblique incidence.

For a light beam that is obliquely incident on a double-layer DOEs substrate (that is, the angle of incidence $\theta_{1}\neq 0$), the diffraction efficiency is related to the microstructure heights, angle of incidence, waveband, and material refractive properties, as follows [12]:

(6) $$\begin{array}{l} \eta_{m}=\sin c^{2}\{m-[\frac{d_{1}\left[\sqrt{n_{M}^{2}\left(\lambda \right)-n_{1}^{2}\left(\lambda \right)\sin^{2}\theta }-n_{1}\left(\lambda \right)\cos\theta \right]}{\lambda }\\ +\frac{d_{2}\left[\sqrt{n_{2}^{2}\left(\lambda \right)-n_{1}^{2}\left(\lambda \right)\sin^{2}\theta }-\sqrt{n_{M}^{2}\left(\lambda \right)-n_{1}^{2}\left(\lambda \right)\sin^{2}\theta }\right]}{\lambda }]\} \end{array}$$

**Figure 5:**
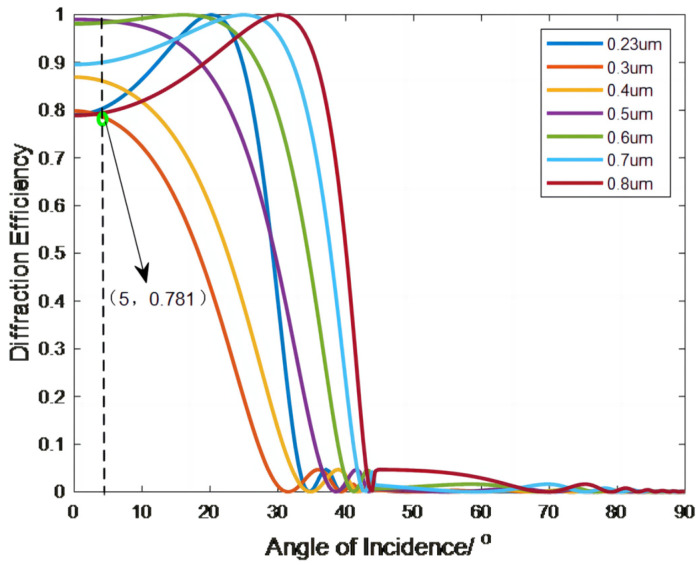
The diffraction efficiency curve of a double-layer DOEs substrate versus the angle of incidence.

CaF_2_ and SiO_2_ are selected for the first and second slice materials, respectively. Air is selected as an intermediate medium material ($n_{M}=1$), and the reference wavelengths are taken as 0.23 μm, 0.3 μm, 0.4 μm, 0.5 μm, 0.6 μm, 0.7 μm, and 0.8 μm. Figure 5 shows the diffraction efficiency of the double-layer DOE versus the angle of incidence.

Figure 5 shows that the double-layer DOE diffraction efficiency is lowest at 0.3 μm and decreases as the angle of incidence increases; for angles of incidence that do not exceed 5°, the diffraction efficiency remains above 78.1%. For angles of incidence exceeding 5°, the diffraction efficiency decreases sharply and drops to 0 at an incidence angle of 30°. This result provides a constraint on the subsequent optical design. To ensure a reasonable diffraction efficiency for the double-layer DOE used in a lens system, the angle of incidence of the light on the lens surface should be controlled to within 5°.

## 3. UV Broad Spectrum Achromatic Lens System Design

The specifications of the design parameters of the lens system are provided in Table 1 below.

**Figure 6:**
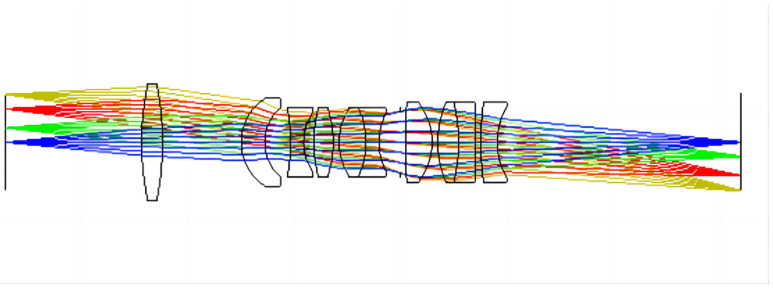
Layout of the refractive lens system.

To ensure the MTF of 30 lp/mm is higher than 0.1 at all wavelengths, the final refractive lens system design consisted of 10 lenses. A diagram of the final optimized structure is shown in Figure 6.

**Figure 7:**
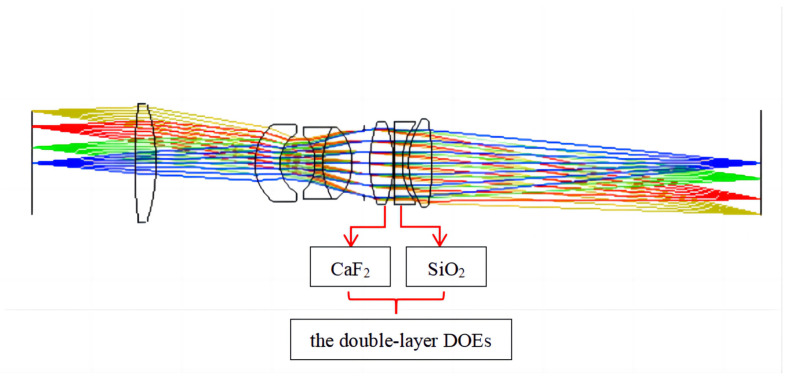
Layout of the refractive/diffractive hybrid lens system.

Using double-layer DOEs in refractive lens systems reduces the number of lenses, improves light transmission by the lens system, and facilitates the fabrication of a lightweight miniaturized lens system. Diffractive surfaces are added to the rear surface of the fifth lens and the front surface of the sixth lens, with CaF_2_ and SiO_2_ as the substrate materials. The angle of incidence of the light affects the diffraction efficiency of double-layer DOEs. In this design, it is necessary to control the incidence angle of the central and edge rays of light on the first slice layer of the double-layer DOEs so that they are 0. The radius of curvature of the lens results in an incident angle for the final edge light on the first substrate of 4.453°, where the diffraction efficiency of the double-layer DOEs at 0.3 μm is higher than 80.54%. Figure 7 shows the structure of the final lens system with seven lenses. The lens data of the refractive/diffractive hybrid lens system are shown in Table 2 and Table 3.

**Figure 8:**
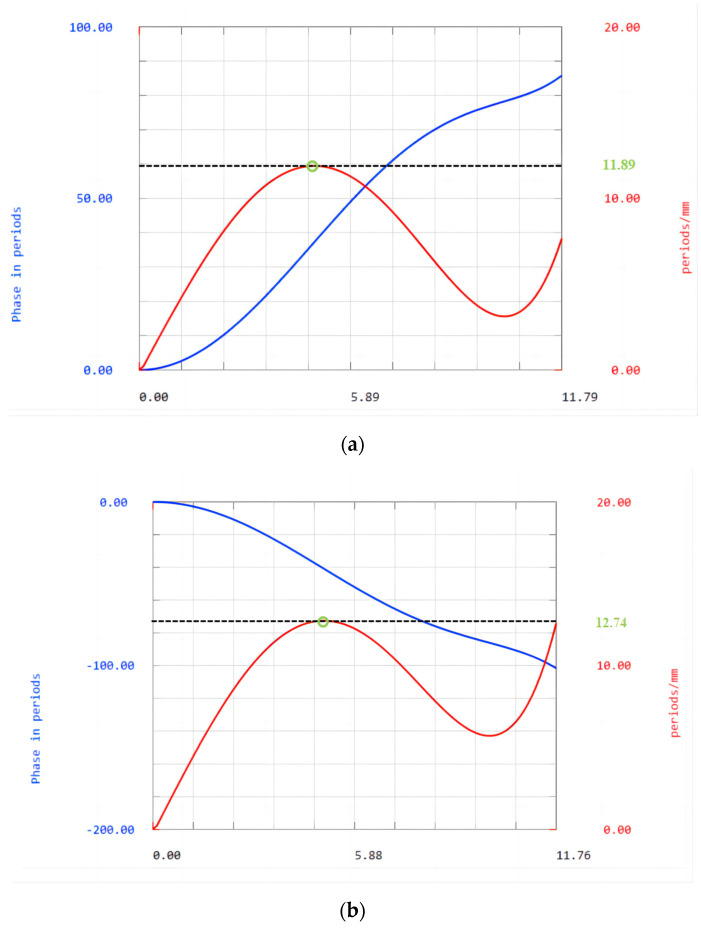
Phase plot of the diffractive surfaces with (**a**) CaF_2_; (**b**) SiO_2_ substrates.

Figure 8 shows a phase plot of the diffractive surface. The abscissa is the diameter; the blue curve represents the additional phase introduced by the binary surface, and the unit is the period; the red curve represents the change of phase at different net diameters (contour frequency), and the unit is cycle per millimeter. The diffractive surfaces with CaF_2_ as substrate have a maximum of 11.89 cycles per millimeter of the ring band, the reciprocal of which corresponds to a minimum cycle width of 84.104 μm. The diffractive surfaces with SiO_2_ as the substrate have a maximum of 12.74 cycles per millimeter of the ring band, the reciprocal of which corresponds to a minimum cycle width of 78.494 μm. The minimum period width of the binary surface used in this study meets the processing requirements. Both binary surfaces can be processed.

The design results for the two lens systems are presented in the figures below.

**Figure 9:**
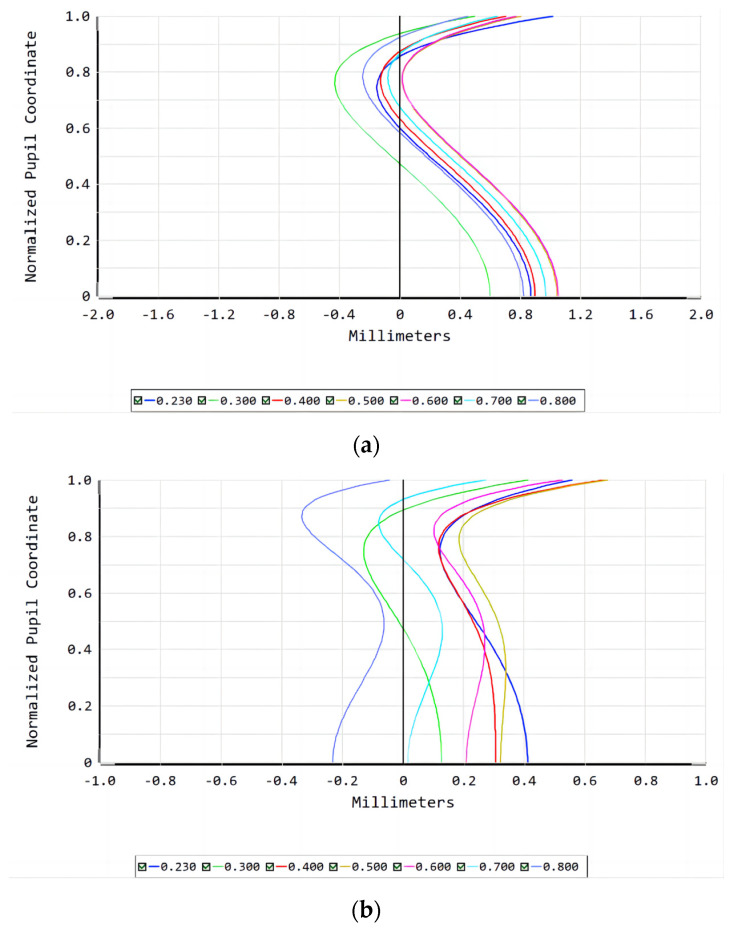
Longitudinal aberration of the (**a**) refractive lens system; (**b**) refractive/diffractive hybrid lens system.

Figure 9 shows the correction of chromatic aberration for the refractive lens and refractive/diffractive hybrid lens systems: the positional chromatic aberration of the refractive/diffractive hybrid lens system is less than 0.4 for an aperture of 0 and less than 1 for an aperture of 1. The positional chromatic aberration for an aperture of 0.7 is less than 0.4.

**Figure 10:**
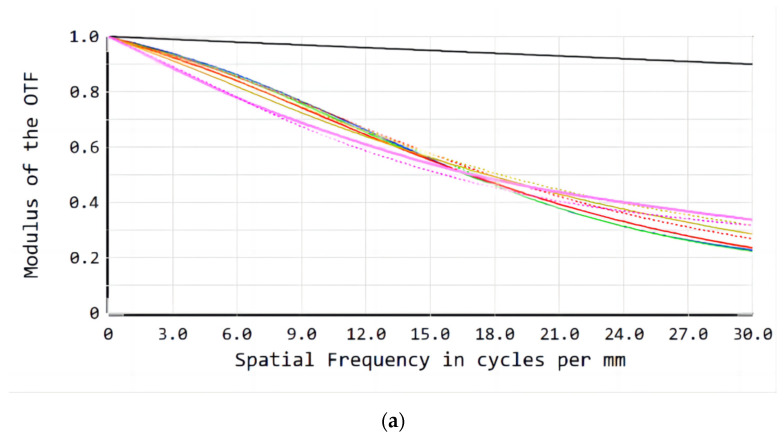

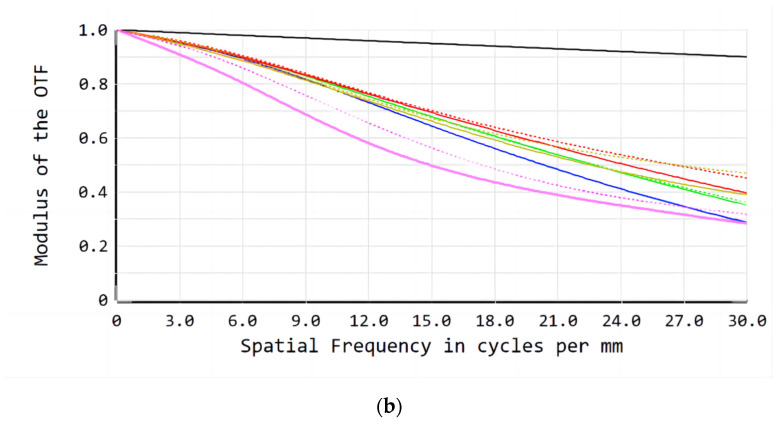
MTF of the (**a**) refractive lens system; (**b**) refractive/diffractive hybrid lens system.

Figure 10 shows the imaging quality results. The MTF of the refractive/diffractive hybrid lens system reaches more than 0.3 at 30 lp/mm and is higher than that of the refractive lens system.

**Figure 11:**
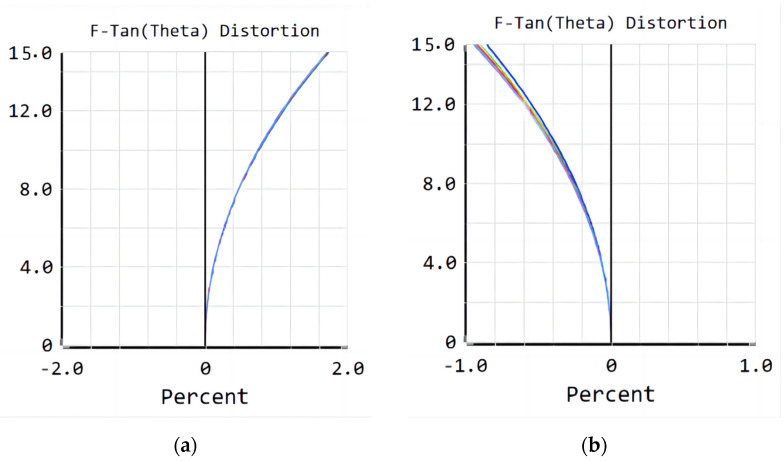
Distortion of the (**a**) refractive lens system; (**b**) refractive/diffractive hybrid lens system.

Figure 11 shows that the maximum distortion of the refractive/diffractive hybrid lens system is −0.95% and the imaging distortion is smaller than that of the refractive lens system during use. The design results show that the optical performance of the refractive/diffractive hybrid lens system is higher than that of the refractive lens system.

According to the data [13], the transmittance at 0.23 μm is 90.61% for 10-mm SiO_2_, 92.06% for 10-mm CaF_2_ and 93.95% for 10-mm MgF_2_. The transmittance of the entire system for the final lens design can be calculated based on the thickness of each lens. The overall transmittance of the refractive lens system is 59.59%. In the final design of the refractive/diffractive hybrid lens system, the incidence angle of light on the first layer of the double-layer DOEs is 4.453°, the minimum diffraction efficiency of the double-layer DOEs is 80.54%, and the overall transmittance of the system is 57.63%. Compared to the refractive lens system, the refractive/diffractive hybrid lens system designed with seven lenses is lighter, simpler and achieves nearly the same transmittance.

## 4. Conclusions

The purpose of this study was to achromatize a UV broad spectrum lens system by using double-layer DOEs. As single-layer DOEs can only be used in narrow bands, double-layer DOEs based on CaF_2_ and SiO_2_ were used in this study. The optimized double-layer DOEs can achieve more than 75% diffraction efficiency in the broad UV spectral range (0.23–0.8 μm). The hybrid refractive/diffractive lens system based on double-layer DOEs achieve a higher image quality (MTF > 0.3 at 30 lp/mm) than the conventional refractive lens system for a similar transmittance, while eliminating the use of three lenses and correcting the positional chromatic aberration more effectively. In addition, the double-layer DOEs designed in this paper can be processed, assembled and adjusted. The hybrid refractive/diffractive lens system is also structurally more effective than the conventional refractive lens system. The hybrid refractive/diffractive lens system provides a feasible solution to the problem of the low transmittance of conventional refractive lens systems over a wide UV spectral range.

## Figures and Tables

**Table 1:** Design parameters of the lens system.

Parameter	Specification
Object height	30 mm (full field of view)
Object space NA	0.1
Focal length	70 mm
Magnification	1
Working band	0.23–0.8 μm
MTF	Above 0.1 at 30 lp/mm

**Table 2:** Lens data for the refractive/diffractive hybrid lens system.

No.	Surf: Type	Radius	Thickness	Material	Clear Semi-Dia
0	OBJECT	Infinity	29.691		15.000
1	Standard	136.572	6.122	CAF2	17.127
2	Standard	−55.787	28.502		17.089
3	Standard	14.565	7.220	F_SILICA	11.148
4	Standard	9.976	10.183		8.585
5	Standard	−11.293	2.198	F_SILICA	8.220
6	Standard	21.079	8.493	MGF2	9.465
7	Standard	−14.245	3.632		10.230
8	STOP	Infinity	1.456		9.366
9	Standard	33.456	6.810	CAF2	11.450
10	**Binary 2**	−31.182	0.140		11.790
11	**Binary 2**	Infinity	2.325	F_SILICA	11.756
12	Standard	18.940	1.909		11.754
13	Standard	22.563	6.781	MGF2	12.419
14	Standard	−64.497	94.909		12.621
15	IMAGE	Infinity	-		14.986

**Table 3:** Diffractive surface coefficient.

No.	Diffraction Order	Maximum Term	Norm Radius	Coeff. on *p*^2	Coeff. on *p*^4	Coeff. on *p*^6
10	1	3	1	12.165	−0.107	3.392 × 10^−4^
11	1	3	1	−12.857	0.111	−3.722 × 10^−4^

## Data Availability

Not applicable.
